# Imaging of hibernomas: A retrospective study on twelve cases

**DOI:** 10.1186/2045-3329-1-3

**Published:** 2011-07-25

**Authors:** Zafiria G Papathanassiou, Marco Alberghini, Sophie Taieb, Costantino Errani, Piero Picci, Daniel Vanel

**Affiliations:** 1 Research, The Rizzoli Institute, Via del Barbiano 1/10, 40106, Bologna, Italy; 2 Pathology C, The Rizzoli Institute, Via del Barbiano 1/10, 40106, Bologna, Italy; 3 Centre Oscar Lambret, Lille, France

## Abstract

**Background:**

To analyze the imaging features of hibernomas on computed tomography (CT) and magnetic resonance (MRI).

**Methods:**

Twelve hibernomas were retrospectively assessed with CT and MR imaging and compared to the histology of the specimen

**Results:**

Nine females and three males with a mean age of 30 years were included. Ten tumors occurred in the thigh and two affected the subcutis of the periscapular and buttock regions. On eight CT scans, seven (87,5%) lesions were homogeneous and mildly to moderately hyperdense compared to subcutaneous fat while one lesion was heterogeneous with mixed hypo and hyperattenuating areas. On six T1W images, five (83,3%) lesions appeared homogeneous and hypointense relative to subcutaneous fat and one was heterogeneous. Incomplete fat suppression was depicted in all cases. All lesions displayed marked enhancement. Large intratumoral vessels were depicted in three of the 12 (25%) cases. Septations were depicted on four of the eight unenhanced CT and on all six MRI examinations.

**Conclusions:**

Hibernoma usually appears hypodense and hypointense relative to subcutaneous fat on pre-contrast CT and MR T1W with variable enhancement patterns and incomplete fat suppression on STIR or fat-saturated sequences. These characteristics relate directly to the presence of brown fat. However, atypical findings such as heterogeneous patterns of mixed fatty and non fatty components on unenhanced CT and MR T1W can be also encountered. Absence of large intratumoral vessels should not exclude hibernomas from the differential diagnosis of regional lipomatous tumors.

## Introduction

Hibernomas are rare benign lipomatous tumors originating from residual brown fat cells. At the beginning of the century, Merkel [1] first described them as"pseudolipomas". Owing to their resemblance to the brown fat of hibernating animals, the term "hibernoma" was coined by Gery in 1914 [2]. They affect chiefly adults in the 3^rd ^of 4^th ^decades of life [3] and they usually grow in the vestiges, where brown fat is found in fetuses and infants, such as the shoulder, neck, axilla, the periscapular and interscapular area, thorax and retroperitoneum [4].

The rareness of this lipomatous tumor and its histologic configuration make it a challenging radiologic diagnosis. To the best of author's knowledge only three series [5-7] and several case reports [8-18] have exhibited the imaging characteristics of hibernomas. The present study, being the largest in the imaging of hibernomas, highlights the spectrum of imaging appearances (CT/MRI) of twelve histologically proven cases of hibernomas and stresses the positive impact of imaging in the pre-operative planning when a complex fatty mass is encountered.

## Materials and methods

Over a 23-year period (1986-2009) fifteen cases diagnosed as hibernomas were identified in the histopathology database of two tertiary referral bone and soft tissue tumor centers. Imaging studies were available in twelve cases. Information regarding age, sex, clinical examination, lesion size and site was registered. Evaluation of the pre-operative imaging investigations (CT-MRI) was performed. Three patients underwent CT and MRI examinations, while five had only CT scans and four had only MRI. Of the latter four patients, two had also ultrasound (U/S) examinations and one of them underwent position emission tomography (^18 ^F FDG-PET). All CT examinations were performed before and after contrast medium intravenous administration. MRI studies obtained from referring institutions included a variety of T1weighted spin-echo (T1WSE), T2weighted spin-echo (T2WSE),T2 weighted fast spin-echo with fat suppression(T2 FSE Fat Sat), short Tau inversion recovery (STIR) and T1W SE with fat suppression sequences(T1 SE Fat Sat). Post gadolinium images were acquired on six cases; one of which had also a MR Angiography. Imaging findings were evaluated by two radiologists (one experienced on bone and soft tissue tumors radiologist and one musculoskeletal radiologist clinical fellow. Radiological assessment included lesion size, location, and internal morphology along with CT attenuation, MR signal intensity and homogeneity, which were compared to subcutaneous fat and muscle. Additionally, contrast enhancement, U/S echogeneity and standard uptake value (SUV) on^18 ^F FDG-PET were recorded. Histopathological analysis was performed by one experienced bone and soft tissue tumor pathologist. All patients had complete but marginal resections of the lesions.

## Results

**Figure 1:**
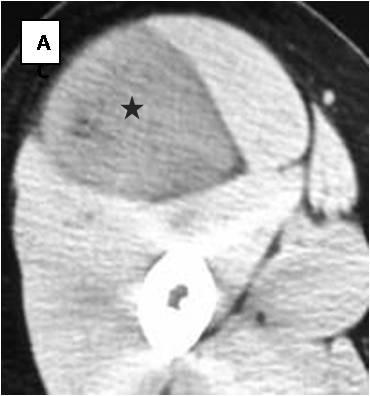
**Unenhanced CT scan (1): A well-defined mass of attenuation close to muscle is located intermuscularly at the anterior aspect of the right upper thigh (asterisk)**.

**Figure 2:**
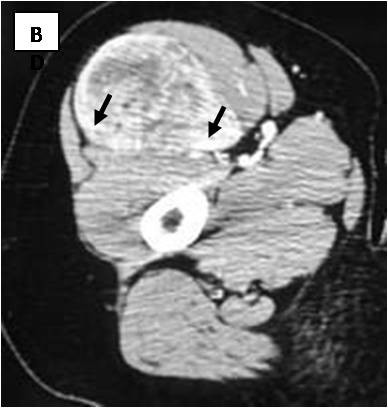
**Axial contrast-enhanced CT scan: Delineation of vessels (black arrows and arrowheads) is apparent on enhanced images**.

**Figure 3:**
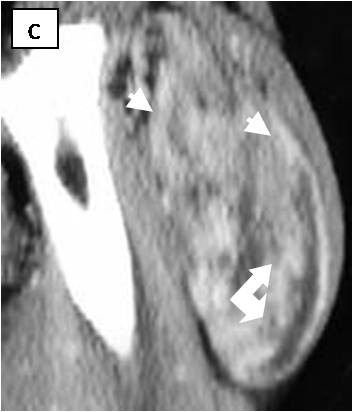
**Sagital contrast-enhanced CT scan**. Vessels are well visible (white arrows and arrowheads)

Table 1 displays the imaging appearances of the presented cases. Of the twelve patients nine were female and three male, from 19 to 46 years old (mean: 30 y). Each patient had one lesion and all of them presented with a slow-growing expansion of the affected soft tissue area. Physical examination revealed palpable lumps of various sizes that were painless and relatively mobile. Laboratory tests were not remarkable. Ten of the twelve lesions were located in the upper thigh (eight in the anterior compartment and two in the posterior compartment) and the other two were located subcutaneously in the lower periscapular and buttock regions. All lesions were well circumscribed and presented with fusiform elongated of ovoid shapes. The smallest lesion measured 5,5 × 4,2 × 1 cm and was located in the left periscapular area and the biggest one measured 24 × 12,7 × 7 cm at the postero-medial aspect of the right thigh. Of the eight lesions examined with CT (Figure 1,2,3) seven were mild to moderate hyperdense compared to subcutaneous fat and hypo to isodense relative to muscle. One lesion was heterogeneous with mixed hypo and hyperattenuating areas. Contrast enhancement was obtained by all (eight) lesions with homogeneous (n = 2) and heterogeneous patterns of enhancement (n = 6). On unenhanced images, internal curvilinear structures, consistent with septations, were identified in four cases and were well delineated on post contrast images. The remaining four lesions, which did not present with septations on pre-contrast exams, clearly demonstrated internal vessels after IV contrast medium administration

**Table 1:** Summary of CT and MRI characteristics of the lesions

Pt no/sex /age(y)	Size (cm)	Location	CT attenuation (pre- cntr)	T1WSE	T2WSE	Fat suppression (T2FSE FS-STIR)	Cntr Enhancement	Lesion Pattern on MRI (Internal Curvilinear **structures**)
1/f/26	9 × 6,5 × 4,1	Rt Thigh	>subc fat	-	-	-	CT/marked heterogeneous	yes

2/f/27	10 × 2 × 4	Rt Thigh	>subc fat	-	<subc fat	-	CT-MRI/marked homogeneous	no

3/f/29	5,5 × 4,2 × 1	Lt Scapula	>subc fat	-	-	-	CT/heterogeneous	yes

4/m/30	15 × 4 × 8,5	Lt Thigh	>subc fat	<subc fat	-	Minimal suppression	CT-MRI/marked heterogeneous	yes(+large vessels)

5/f/34	8 × 7 × 3	Lt Thigh	-	<subc fat	-	-	-	yes

6/m/46	24 × 12,7 × 7	Rt Thigh	Heterogeneous	Heterogeneous	Heterogeneous hyperintense	Partial suppression	CT-MRI/marked heterogeneous	yes(+large vessels)

7/f/19	8 × 6 × 2	Rt Thigh	>subc fat	-	-	-	CT/homogeneous	yes

8/m/31	11 × 7 × 4,2	Rt Thigh	>subc fat	-	-	-	CT/marked heterogeneous	yes

9/f/17	17 × 9 × 4	Rt Thigh	>subc fat	-	-	-	CT/marked heterogeneous	no

10/f/39	9,4 × 5,9 × 4,9	Rt Thigh	-	<subc fat	<subc fat	Partial suppression	MRI/marked heterogeneous	yes

11/f/31	6 × 4 × 4,5	Lt Buttock	-	<subc fat	-	Minimal suppression	MRI/marked heterogeneous	yes (+large vessels)

12/f/23	10 × 6 × 6	Lt Thigh	-	<subc fat	<subc fat	Minimal suppression	MRI/marked homogeneous	yes

**Figure 4:**
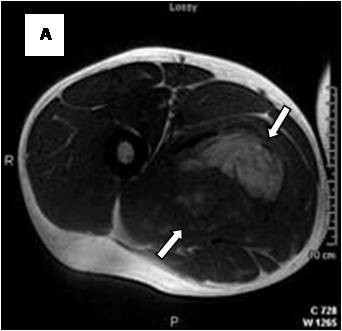
**Axial T1WSE**.

**Figure 5:**
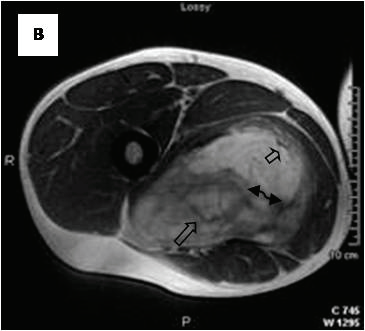
**Axial T2WSE The mass contains ill-defined areas of lower intensity relative to subcutaneous fat on T2WSE**.Internal septations are evident (curved double arrow).

**Figure 6:**
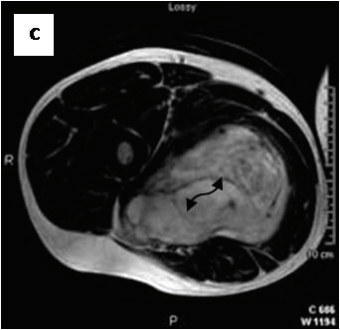
**Post gadolinium image**. The mass of heterogeneous mixed intensities exhibits diffuse enhancement. Unenhanced curvilinear septations are well visible (curved double arrow).

**Figure 7:**
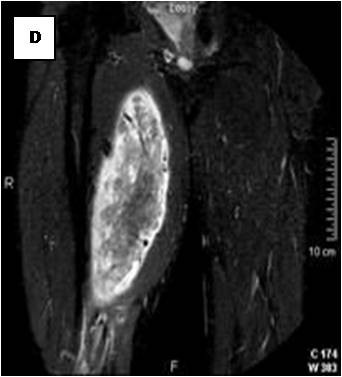
**Partial loss of fat signal intensity is depicted on STIR images**.

**Figure 8:**
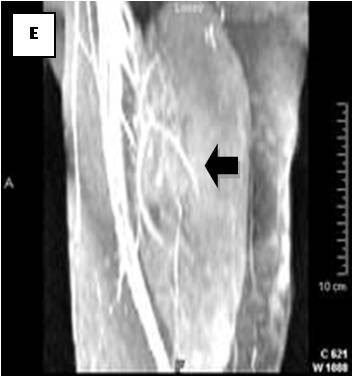
**Sagital reformatted image clearly exhibits large intratumoral vessels (black arrows)**.

**Figure 9:**
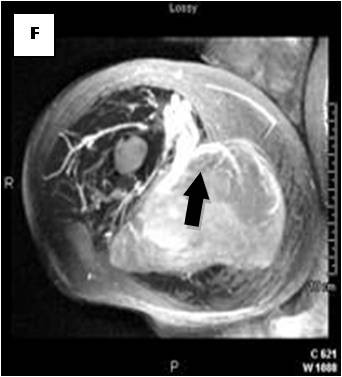
**Axial reformatted image**.

**Figure 10:**
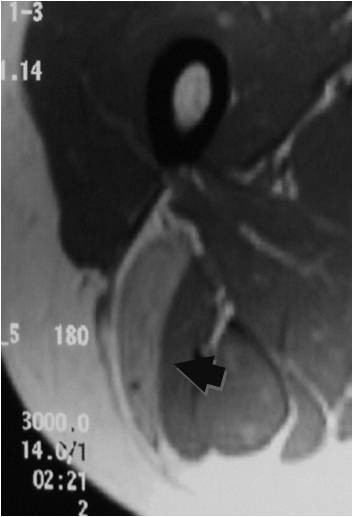
**Axial PDWSE exhibits an intermuscular soft tissue mass that is hypointense relative to subcutaneous fat (arrow)**.

**Figure 11:**
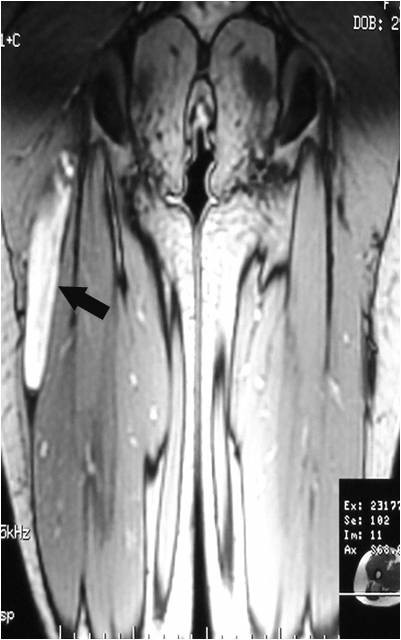
**Homogeneous enhancement is observed (arrow)**.

**Figure 12:**
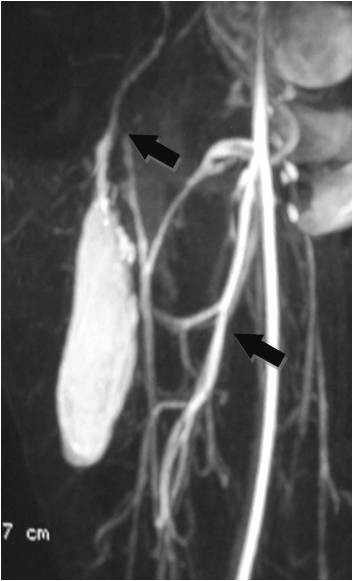
**On MRA, blood supply is originated from epigastric and deep femoral vessels (arrows)**.

**Figure 13:**
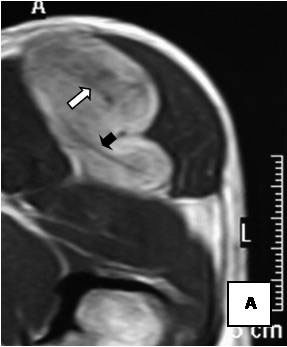
**Axial T1WSE before injection**.

**Figure 14:**
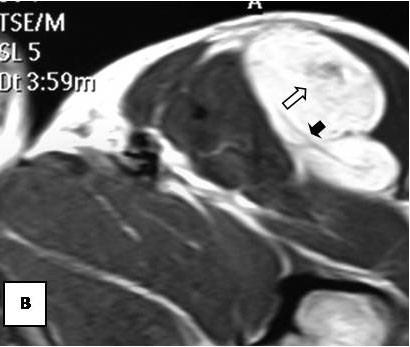
**Axial T1WSE after injection enhanced (white arrows) and unenhanced (black arrows) thin curvilinear structures corresponding to fibrovascular and fibrous tissue, respectively**.

**Figure 15:**
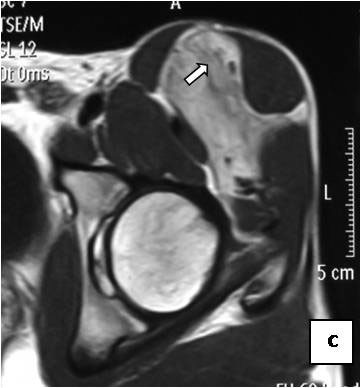
**Axial T1WSE before injection at another level**.

**Figure 16:**
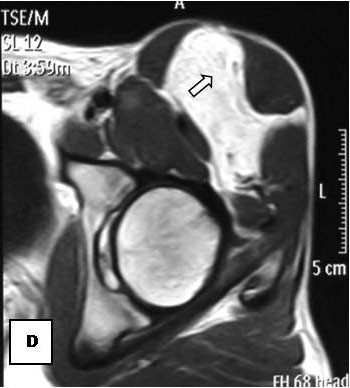
**The same level after injection**.

**Figure 17:**
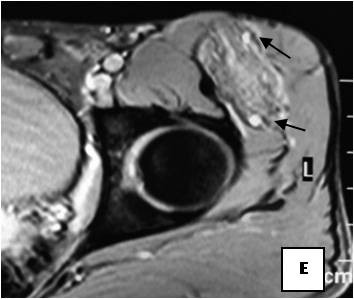
**On T2 GRE sequence, internal thin vessels are also seen (thin black arrows)**.

**Figure 18:**
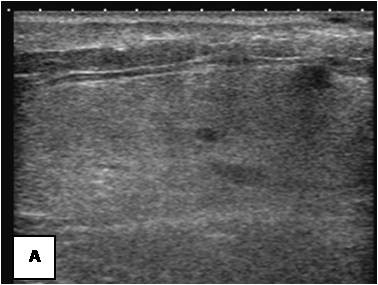
**Ultrasonography exhibits a mild heterogeneous hyperechoic mass**.

**Figure 19:**
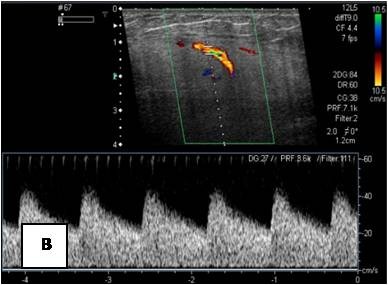
**It contains prominent vessels with Doppler**.

**Figure 20:**
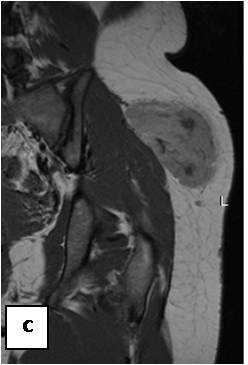
**T1WSE: subcutaneous mass of the lateral aspect of the left buttock that is clearly hypointense to subcutaneous fat**.

**Figure 21:**
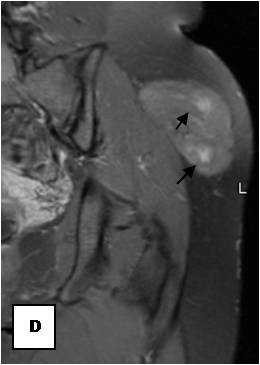
**T1WSE FAT SAT: the lesion is poorly pre saturated**.

**Figure 22:**
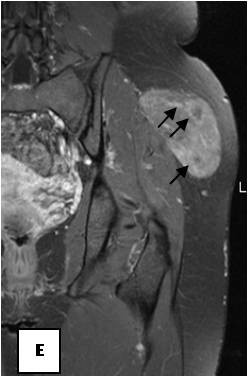
**T1WSE FAT SAT with gadolinium: the lesion contains vessels of various sizes (black arrows)**.

**Figure 23:**
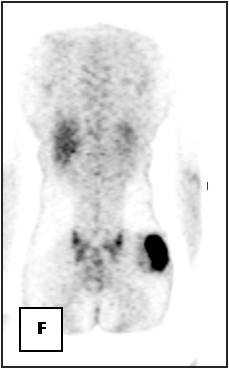
**On^18 ^F FDG-PET scan, the lesion has shown increased FDG accumulation**.

**Figure 24:**
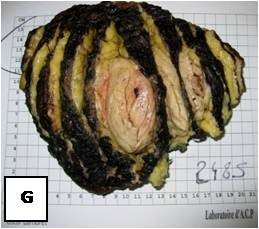
**Gross surgical specimen reveals an encapsulated, lobular mass with yellow-tan to dark brown cut surfaces**.

**Figure 25:**
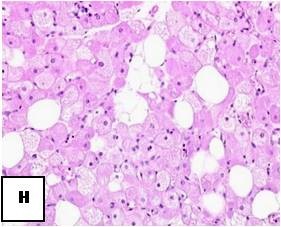
**Hematoxylin-Eosin stain: Multiple multivacuolated cells are identified with some scattered white adipocytes**.

**Figure 26:**
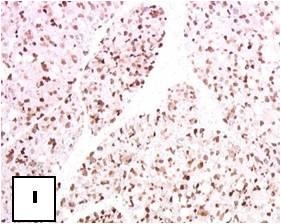
**Immunohistochemistry: S-100 positivity is evident**.

On T1-weighted images, five lesions appeared slightly hypointense relative to subcutaneous fat and hyperintense compared to muscle while the largest tumor showed heterogeneous-mixed intensity with components of increased and decreased intensity (Figure 4,5,6,7,8,9). Three out of four lesions examined with T2-weighted sequences, presented with slightly hypointense masses compared to subcutaneous fat and one was heterogeneously hyperintense. On STIR and T2 fat sat sequences, only minimal to partial signal loss was depicted (Figure 7) in all cases. One patient, who had additionally a MR angiography exhibited rich vascularity of the lesion as well as the origin of the blood supply from the epigastric and deep femoral vessels (Figure 10,11,12). Post gadolinium images (T1WSE/T1 SE Fat Sat) revealed marked heterogeneous enhancement in four lesions and marked homogeneous in two lesions. Internal curvilinear and branching structures of low signal intensity on T1WSE and T2WSE were shown in all six cases (Figure 13,14,15,16,17). Gadolinium uptake was not visible in all curvilinear strands (Figure 2B, 4A-D). On the other hand, post gadolinium visualization of vessels was noticed in all six cases. Intratumoral vessels of larger caliber were detected in three of the 12 (25%) cases. (Figure 2E-F,4E,5D-E). The sonographic appearance of the two lesions was that of a heterogeneous hyperechoic mass containing prominent vasculature (Figure 18,19). On^18 ^F FDG-PET scan, the subcutaneous lesion at the left buttock presented with an increased SUV value (Figure 20,21,22,23,24,25,26). All patients experienced an uneventful post-surgical recovery. No case relapsed.

## Discussion

Hibernomas are rare slow-growing benign tumors that consist of brown fat. In 1670, Welch [19] was the first to describe this specialized form of adipose tissue in hibernating animals. None the less, brown fat is also found in more than fifty nonhibernating species, such as human fetuses and newborns [20]. It is believed to represent a kind of fetal fat whose function is to promote nonshivering thermogenesis and gradually is replaced by white adipose tissue with advancing postnatal age to finally comprise less than 0, 1% of the total body weight by the age of 70 years [4, 17, and 21]. However, it may persist in various portions throughout adulthood [9]. Hibernoma is the only tumor known to occur within brown fat and can grow at any location where brown fat remains [6,16]. Most commonly hibernomas form in the vestiges where brown fat has remained from fetal life such as the periscapular and interscapular region, the neck, axilla, mediastinum, upper thorax and retroperitoneum [4,22,23]. Other uncommon locations include the abdomen, thigh, buttock, popliteal fossa and intracranial sites [4]. Based on the largest and most valid demographic study (Soft Tissue AFIP Registry), by Furlong MA et al [3], hibernomas affect mainly adults in the 3^rd ^and 4^th ^decades of life (61% of cases) with a mean age of 38 years. Unlike the previous published data, the AFIP series [3] demonstrates a slight male predominance (58% of cases) with the thigh being the most common location (30% of cases). Our study results are consistent with the aforementioned findings regarding age (range: 19-46 y, mean: 30 y) and location (83,3% of cases located in the thigh) but on the other hand a clear female predilection (75% of cases) is shown in this series.

Generally hibernomas exhibit a rather quiet clinical behavior and present as slow growing soft tissue masses that are usually painless and relative mobile. Owing to the tumor's hypervascularity, localized warmth can be depicted over the lesion at palpation [4,6,7,14,15]. The lesions can become symptomatic when compression of nearby structures occurs [6,15]. No evidence of a malignant form of hibernoma has been reported in the English literature, except for the case published as an abstract by Teplitz et al. [24] that involved a sarcoma with hibernoma-like features. Incomplete excision results in local recurrence of the tumor; therefore marginal but complete resection is considered as the treatment of choice for these lesions [14,24]. Even though core needle biopsy is not recommended in cases of suspected hibernoma due to the tumor's hypervascularity [9,14,25] all of the presented cases were preoperatively biopsied without any complications. From a macroscopic aspect, hibernomas are well-defined, encapsulated soft, lobulated masses and the color ranges from tan to red brown [15] (Figure 4G.). They usually measure from 5 to 10 cm in diameter, but they may reach up to 20 cm [4,15]. Microscopically, the tumor is characterized by multivacuolated cells with eccentric nuclei and granular eosinophilic cytoplasm, univacuolated cells with peripheral nuclei, and smaller round cells with granular cytoplasm. The hypervascularity and the presence of cells with eosinophilic granular cytoplasm full of mitochondria give hibernomas their brown color [4,6,18]. From an histological point of view this entity must be distinguished from granular cell tumor, that is a benign peripheral nerve derived tumor composed of granular cells rich in mitochondria. In this regard immunohistochemistry does not help, because both tumors intensely stain for S-100 protein. The main histological difference is that hibernoma shows much more pleomorphism and focally show typical mature adipocytes, in between the granular cells. The diagnosis of lipomatoustumors is often very difficult. Molecular pathology can better classify these lesions and made past classifications out of date. But cytogenetics studies do not help in the diagnosis of hibernoma [26].

According to the 2002 WHO classification there are six histologic subtypes of hibernomas [27]. These are only of diagnostic relevance and not of prognostic value. Histopathologic evaluation of hibernomas, as previously described, is well-established and pathognomonic. On the contrary, CT and MRI features are not specific and vary with the nature and amount of lipid component [4,12,18,19,22,23]. Non contrast CT usually demonstrates a well-demarcated soft tissue mass of predominantly low attenuation which is close but not identical to subcutaneous fat. On the other hand, more heterogeneous patterns can be encountered as well, as in this series. Internal linear, curvilinear or branching septations-like densities may be contained [7,9,10]. On post contrast scans, enhancement of the septa as well as more diffuse uptake, usually occurs [7, 9, and 23]. Diffuse enhancement was depicted in all the present cases whereas internal enhancing linear or curvilinear densities were shown in four out of eight cases, indicating thus internal vasculature. Even though vessels were shown in the remaining four cases on post contrast images; the absence of septations in these lesions prior to contrast infusion was attributed to the fact that these lesions had attenuations closer to muscle than fat. On MR images, as in previously published data [6-16,18], five out of six lesions presented, on T1WSE sequences, slightly to moderately decreased signal intensity relatively to subcutaneous fat and only one showed a heterogeneous-mixed signal intensity including areas of increased and decreased intensity but on the whole slightly lower than subcutaneous fat, probably due to a greater "hibernoma" component. Three lesions on T2WSE images demonstrated slightly lower intensities than subcutaneous fat; although most authors report signal intensities closer to fat [5,7,9,11-14]. The heterogeneous lesion on T1WSE remained heterogeneously hyperintense on T2WSE images as well. Finally, like in most cases [5-7,13-17], STIR and T2 fat sat sequences failed to achieve full suppression of the examined hibernomas and displayed the most heterogeneous patterns. Gadolinium enhancement, either heterogeneous or homogeneous, is usually present in hibernomas [5,7,11-18]; even though Cook M et al [8] and Lee J [6] et al did not report any significant gadolinium uptake in their cases. Although, internal curvilinear structures of low signal intensity were observed on T1WSE and T2WSE sequences in all lesions, they didn't exhibit the same degree of enhancement most likely corresponding to hypocellular fibrous and fibrovascular tissue interspersed with the fatty and non fatty portions of the tumor [6,14]. Little is known regarding the imaging of hibernomas on^18 ^F FDG-PET scans. The reported high FDG accumulation in these fat-containing tumors may be attributed to the metabolically active cellular elements rather than reflect their malignant or not potential [28,29].

Various differential considerations, based on imaging, can be suggested when a complex fatty mass is encountered, including benign entities like lipoma, angiolipoma and hemangioma as well as malignant tumors like liposarcoma. Lipomas present as homogeneous fatty masses with few scattered internal septa and no signs of enhancement [6]. Angiolipomas and hemangiomas can be distinguished in terms of different morphology of internal vasculature [13,16,17]. Several studies [4,16-18] stress the importance of large branching intratumoral vessels with early contrast enhancement and AV shunting in the differential diagnosis of hibernomas. However these features are not always present, although fine enhancing strands may be seen [6]. In the present series, internal vessels were apparent in six MRI exams; while in total three lesions contained vessels of larger caliber as well. So, vascularity either in the form of thin enhancing septa or in the form of vessels is primarily anticipated in hibernomas. On the other hand, absence of large intratumoral vessels should not exclude hibernomas from the differential diagnosis. Well-differentiated liposarcomas are characterized by the presence of irregularly thick (>2 mm) and/or nodular septa, foci of high T2 and prominent areas of enhancement [6,15]. Moreover, the fatty component of a well-differentiated liposarcoma appears isointense to subcutaneous fat, on T1WSE; distinguishing them from hibernomas [6]. Other lesions like myxoid liposarcoma and clear cell sarcoma could be similar to brown fat tumors but the former displays intense heterogeneity on T2 sequences and the latter primarily involves a tendon, ligament or aponeurosis [13].

This study has limitations, such as limited number of cases, and examinations performed with different techniques. None the less, this study comprises the largest number of cases of this rare tumor published thus far and elaborates effectively on its various imaging appearances. Conclusively, even if CT and MRI features are not specific, hibernoma should be strongly suggested if a soft tissue mass, exhibits higher attenuation than subcutaneous fat on CT, slightly lower signal intensity relative to subcutaneous fat on T1WSE, marked enhancement and partial fat suppression on STIR and fat-saturated sequences. These differences compared to subcutaneous fat, especially on MRI, reflect the different nature of lipid component of hibernomas and comprise the cornerstone in differentiating them from malignant lipomatous tumors. However, as in this study, other atypical findings such as more heterogeneous patterns of mixed fatty and non fatty components on unenhanced CT and MR T1W may be encountered. Furthermore internal septations, regardless of enhancement, and thin vessels contribute in establishing the diagnosis. The role of large intratumoral vessels remains questionable in characterizing hibernomas. While complete surgical resection is curative for hibernomas, knowledge of its MRI and CT features can help narrow the field of differential diagnosis and modify adequately the pre-operative planning of complex lipomatous tumors.

## Competing interests

The authors declare that they have no competing interests.

## Authors' contributions

All authors have read and approved the final manuscript.

ZP looked at the cases and wrote the article, MA checked the histology, and the text, ST gave one case and checked the text, CE checked the surgical part, PP checked the research part, DV proposed the article, reviewed the cases and checked the text.
